# Assessment of self-Esteem and academic stress among nursing students

**DOI:** 10.6026/9732063002001886

**Published:** 2024-12-31

**Authors:** Buvaneswari Ramakrishnan, Devikrishna R., Mahalakshmi B., Monisha M., Baskaran M., Siva Subramanian N., Jeya Beulah D.

**Affiliations:** 1 Department of Psychiatric Nursing, KMCH College of Nursing, Coimbatore, Tamil Nadu - 641048,India; 2 Department of Pediatric Nursing, Nootan College of Nursing, Sankalchand Patel University, Visnagar, Gujarat - 384315, India; 3 Department of Psychiatric Nursing, Shree Venkateshwara College of Nursing, Erode,Tamil Nadu - 638455, India; 4 Department of Psychiatric Nursing, PSG college of Nursing, Coimbatore, Tamil Nadu - 641004, India; 5 Department of Psychiatric Nursing, Nootan College of Nursing, Sankalchand Patel University, Visnagar, Gujarat - 384315, India; 6 Department of Community health Nursing, SCPM College of Nursing & Paramedical Sciences, Gonda, Uttarpradesh-271003, India

**Keywords:** Self-esteem, academic stress, nursing students, rosenberg self-esteem scale

## Abstract

Self-esteem is a crucial psychological factor influencing how individuals handle stress, particularly in challenging academic settings. This study aimed to assess the relationship between self-esteem and academic stress among nursing students. A quantitative, cross-sectional descriptive design was employed, with 243 nursing students from various year levels at College of Nursing in Puducherry. Data were collected using the Rosenberg Self-Esteem Scale (RSES) and the Scale for Assessing Academic Stress (SAAS). Descriptive and inferential statistics, including chi-square tests, were used for analysis. Indicates that 66.7% of students exhibited normal self-esteem, while 50.2% experienced moderate academic stress. Significant associations were found between academic stress and factors such as age, year of study and place of stay. Notably, senior students reported higher stress and lower self-esteem.

## Background:

Self-esteem, a fundamental aspect of an individual's psychological health, refers to the overall evaluation of one's worth or value [1]. It plays a crucial role in shaping how individuals manage stressful situations, particularly in demanding environments such as academic institutions. Nursing students, in particular, face a unique set of stressors due to the dual demands of academic coursework and clinical training [2]. High self-esteem helps students manage stress effectively, fostering resilience and improving coping mechanisms, while low self-esteem can exacerbate stress and negatively impact academic performance and mental health [3]. Academic stress, often described as the mental distress related to anticipate academic failure or the pressure to succeed, is common among students, particularly in high-demand fields like nursing. Students in nursing programs are faced with multiple challenges, including heavy coursework, exams, clinical responsibilities and high expectations from both educators and families. Research shows that excessive academic stress can lead to negative outcomes such as burnout, anxiety and even depression, further contributing to a decline in self-esteem [4]. Several studies have demonstrated the close relationship between self-esteem and academic stress. Yildirim *et al.* found that nursing students with higher self-esteem coped more effectively with academic and clinical stressors [5]. Similarly, Acharya Pandey and Chalise observed that 78% of nursing students had low self-esteem, while 74% experienced high academic stress, demonstrating the significant overlap between these issues [6]. This study aims to assess the levels of self-esteem and academic stress among nursing students and explore the relationship between these factors. The results will help in understanding how nursing students cope with stress and how self-esteem can influence their academic experiences.

## Methodology:

## Research design:

This study utilized a quantitative, cross-sectional descriptive research design to assess self-esteem and academic stress levels among nursing students [6, 7] . This design allowed for the collection of data without manipulating any variables, providing a snapshot of the participants' current psychological and academic states.

## Setting:

The research was conducted at the College of Nursing in Puducherry, which offers B.Sc. Nursing, M.Sc. Nursing and Post-Basic Diploma programs. The college provides a conductive environment for both academic learning and clinical training, making it an ideal setting for this study.

## Participants:

A total of 243 nursing students from different year levels of B.Sc., M.Sc. and Post-Basic Diploma programs were selected through stratified sampling. Inclusion criteria included nursing students aged 18 years and above, those willing to participate and those available during data collection. Exclusion criteria involved students who were acutely ill during the study period.

## Instruments:

Data were collected using a structured questionnaire that included socio-demographic information and two standardized tools. The Rosenberg Self-Esteem Scale (RSES) was used to assess self-esteem, consisting of 10 items rated on a 4-point Likert scale. The Scale for Assessing Academic Stress (SAAS) was used to measure academic stress levels, containing 30 yes/no questions related to various stress indicators.

## Data analysis:

Descriptive statistics (mean, standard deviation) and inferential statistics (chi-square tests, t-tests) were used to analyze the data. The chi-square test was used to examine the association between self-esteem and academic stress with demographic factors such as age, gender, year of study and place of stay. Statistical analysis was conducted using SPSS version 20 and significance was set at p<0.05.

## Result:

Table 1 illustrates the demographic breakdown of the study participants, showing that the majority were aged 20-25 years (62.6%), predominantly female (84.8%) and stayed at home (57.6%). Such characteristics provide a foundation for understanding how demographic factors might influence academic stress and self-esteem levels. Table 2 highlights significant associations, such as age and place of stay with academic stress (p < 0.05) and gender and year of study with self-esteem (p < 0.05). These findings reinforce the importance of addressing demographic influences when implementing stress-reduction programs for nursing students. The bar graph demonstrates that most nursing students experienced moderate academic stress (50.8%), with fewer reporting slight (31.4%) or high stress (12.7%). This highlights the prevalence of moderate stress levels in nursing students. Figure 1 (see PDF) Distribution of participants based on levels of academic stress, categorized into slight, moderate and high stress. Most students experienced moderate stress (50.8%), with fewer reporting slight stress (31.4%) or high stress (12.7%). Figure 2 (see PDF) shows that 83.9% of nursing students had high self-esteem, whereas only 16.1% exhibited normal self-esteem. This suggests that despite academic stress, most students maintain a positive self-perception.

## Discussion:

This study aimed to assess the relationship between self-esteem and academic stress among nursing students, highlighting the impact of demographic factors such as age, gender, year of study and place of stay. Our findings revealed that while most students (66.7%) had normal self-esteem, a significant portion (50.2%) experienced moderate levels of academic stress, consistent with existing literature. The prevalence of moderate academic stress among students aligns with Yildirim *et al.* who found that nursing students face significant stress due to the demands of clinical training and academics [5]. Similarly, Edwards *et al.* reported that stress peaks during the final year of nursing programs, which corresponds with our finding that higher-year students experienced increased stress levels [9]. Our results indicate a negative relationship between self-esteem and academic stress, which supports the findings of Acharya Pandey and Chalise [8]. They observed that students with low self-esteem were more likely to suffer from high academic stress, reinforcing the importance of self-esteem as a protective factor in managing stress. Gender differences in self-esteem were observed, with female students showing slightly higher self-esteem than male students, a result supported by Golan *et al.* [10]. However, no significant gender differences in academic stress were found, similar to the findings of Rahardjo. In contrast, Verma reported that female students tend to exhibit greater behavioural responses to stress, suggesting that while stress levels may be similar, its manifestations could vary by gender [11]. Our findings align with studies emphasizing the link between academic stress and self-esteem in nursing students. Acharya Pandey and Chalise (2015) reported high academic stress in 74% of students and low self-esteem in 78%, highlighting the need for targeted interventions [12]. Similarly, Uma (2023) found significant associations between academic stress, self-esteem and demographic factors [13]. Jirel *et al.* (2023) noted moderate academic stress in 50.8% of students but high self-esteem in 83.9%, suggesting varied coping mechanisms [14]. These findings stress the importance of counselling, stress-reduction measures and skills development to improve resilience and well-being among nursing students. Our study also found significant associations between year of study and both academic stress and self-esteem, with higher stress and lower self-esteem reported among senior students. Nguyen *et al.* similarly noted that increased academic demands in later years contribute to higher stress levels and declining self-esteem [15]. Place of stay was significantly associated with academic stress but not with self-esteem. Students living at home experienced slightly higher stress levels, which could be attributed to additional responsibilities at home, as suggested by Alkhawaldeh [16]. In conclusion, this study underscores the need for interventions aimed at improving self-esteem and managing academic stress in nursing students. Initiatives such as counseling and peer support programs can help students cope more effectively with the demands of their academic and clinical training.

## Figures and Tables

**Table 1:** Demographic characteristics of nursing students (N = 243)

**Variable**	**Frequency (N)**	**Percentage (%)**
**Age**		
Below 20 years	85	35
20-25 years	152	62.6
Above 25 years	6	2.4
**Gender**		
Male	37	15.2
Female	206	84.8
**Year of Study**		
B.Sc. First year	45	18.5
B.Sc. Second year	67	27.6
B.Sc. Third year	52	21.4
B.Sc. Fourth year	37	15.2
M.Sc. First year	22	9.1
M.Sc. Second year	12	4.9
Post-Basic Diploma	8	3.3
**Place of Stay**		
Hostel	103	42.4
Home	140	57.6

**Table 2:** Association between demographic variables, academic stress and self-esteem (N = 243)

**Demographic Variables**	**df**	**Chi-Square Value (χ^2^) for Academic Stress**	p-value (Academic Stress)	Chi-Square Value (χ^2^) for Self-Esteem	p-value (Self-Esteem)
Age	4	11	0.026*	0.229	0.892
Gender	1	0.053	0.974	6.12	0.032*
Year of Study	12	32.3	0.001**	6.98	0.021*
Place of Stay	2	11.7	0.003*	1.42	0.146
*-p<0.05 significant
*-p<0.001 highly significant
